# Inhibitory Performance in Smokers Relative to Nonsmokers When Exposed to Neutral, Smoking- and Money-Related Pictures

**DOI:** 10.3390/bs11100128

**Published:** 2021-09-22

**Authors:** Afework Tsegaye, Cuiling Guo, Renáta Cserjési, Leon Kenemans, Gijsbert Stoet, Gyöngyi Kökönyei, Alexander Logemann

**Affiliations:** 1 Doctoral School of Psychology ELTE, Eötvös Loránd University, 1064 Budapest, Hungary; tafeworkpsy@gmail.com (A.T.); cuiling0810@gmail.com (C.G.); 2 Institute of Psychology ELTE, Eötvös Loránd University, 1064 Budapest, Hungary; cserjesi.renata@ppk.elte.hu (R.C.); kokonyei.gyongyi@ppk.elte.hu (G.K.); 3 Department of Experimental Psychology, Helmholtz Institute, Utrecht University, 3584 CS Utrecht, The Netherlands; J.L.Kenemans@uu.nl; 4 Department of Psychology, University of Essex, Colchester C04 3SQ, UK; g.stoet@essex.ac.uk

**Keywords:** smoking, nicotine, addiction, inhibition, impulsivity, reward

## Abstract

Introduction: Smoking is associated with significant negative health consequences. It has been suggested that deficient inhibitory control may be implicated in (nicotine) addiction, but its exact role has not yet been elucidated. In the current study, our aim was to investigate the role of inhibitory control in relation to nicotine addiction in contexts that differ in terms of reward. Methods: Participants filled out questionnaires and performed a go/no-go task with three conditions. In one condition, the stimuli were neutral color squares, and in the reward conditions, these were smoking-related pictures and money-related pictures, respectively. In total, 43 non-abstinent individuals that smoke and 35 individuals that do not smoke were included in the sample. Results: The main results showed that individuals that smoke, relative to individuals that do not smoke, had reduced inhibitory control in both reward contexts, relative to a neutral context. The reductions in inhibitory control were mirrored by speeded responses. Conclusions: Individuals that smoke seem to present with reduced inhibitory control, which is most pronounced in contexts of reward. Consistent with incentive sensitization theory, the reduced inhibitory control may be (at least partly) due to the heightened approach bias to reward-related stimuli as indicated by the speeded responses.

## 1. Introduction

It is commonly known that smoking represents a serious public health problem; approximately eight million people die of smoking-related diseases each year [1]. Even in light of this common knowledge, the global prevalence of tobacco smoking in adults is around 20% [1]. This underscores the need for a more thorough understanding of the mechanism that accounts for the persistence of smoking behavior. Perhaps not surprisingly, previous studies suggest that inhibitory control may be impaired in individuals that smoke [2,3], though some studies do not suggest such a clear relation [4]. As suggested by Luijten et al. [3], the relationship may depend on the reward context. However, the exact role of the reward context on the relation between inhibitory control and nicotine addiction has not yet been thoroughly explored.

Inhibitory control can be defined as the general ability of an individual to suppress a planned or prepotent response and is commonly assessed, using the stop signal task and go/no-go task [5,6]. Inhibitory control is important in everyday functioning; deficits of inhibitory control have been reported in various addictions, such as cocaine addiction [7], nicotine dependence [3,8], alcohol dependence [9,10,11], and methamphetamine abuse [12]. It has recently been shown that deficits of inhibitory control are also implicated in behavioral addiction, such as internet addiction [13] and obesity, characterized by high body mass index (BMI) [14,15,16]. Indeed, there seems to be sizeable overlap in terms of the brain mechanism and behavioral manifestation implicated in both pharmacological and non-pharmacological addictions [17,18].

With respect to the mechanism, addiction is associated with a disruption of the dopamine motive system as postulated by Volkow et al. [17]. To elaborate, on the biological level, motivation and associated approach behavior are driven (at least in part) by striatal dopaminergic (DA) neurotransmission. Generally, the percept of stimuli that are associated with any type of reward (e.g., palatable food) increases striatal DA neurotransmission, and increases the chance of approach behavior toward the reward [19]. As mentioned, this dopamine motive system may be dysregulated in addicted individuals, due to repeated exposure to potent rewards. Specifically, in addiction the repeated exposure to reward may result in an overactivation of the DA system and sensitization to reward-related stimuli as well as a net downregulation of striatal dopamine D2 receptors [17,20]. This is congruent with the incentive sensitization (IS) theory as postulated by Robinson [18]. Similarly, IS theory entails that repeated exposure to rewarding substance(s) results in heightened sensitivity to these drugs and associated stimuli, which in turn is related to heightened motivation or approach bias to reward-related stimuli. This is relevant in relation to inhibition, as increased approach tendencies may challenge inhibitory performance. In addition, it should be noted that the striatum is not only important for reward processing, but striatal D2 drives (at least in part) inhibitory control [21].

Taken together, it follows that the mentioned inhibitory deficits may be more pronounced in a context of reward (at least partly), due to higher response tendencies in such conditions. It is also important to note that IS theory predicts cross-sensitization in that heightened sensitization to one drug or a reward-related stimulus may extend to other rewards [18]. This notion is further supported by the known comorbidity between nicotine addiction and addiction to other substances [22]. Hence, it may be suggested that the inhibitory deficits may extend to other conditions of reward.

The relation between nicotine addiction and inhibitory control in a smoking context relative to a neutral context is addressed, at least partly, with a go/no-go task [3,23]. In short, in a standard go/no-go task, go stimuli and no-go stimuli are sequentially presented in random order. Go stimuli require a simple response (i.e., spacebar) and no-go stimuli require a response to be withheld [6]. The relevant outcome variable is the proportion of inhibitions (number of successful inhibitions in no-go trials, divided by the number of no-go trials). Although this measure is plausibly affected by response tendencies, it is thought to reflect (at least partly) inhibitory control [6].

In the go/no-go task implementation of Luijten et al. [3], smoking-related go and no-go stimuli were included in addition to neutral go/no-go stimuli. The results showed that smokers relative to nonsmokers presented with an overall reduced proportion of inhibitions in no-go trials. However, the stimulus type (neutral/smoking related) did not moderate the relationship. In other words, the results did not confirm that individuals that smoke, relative to those that do not smoke, presented with a more pronounced deficit in inhibitory control in the smoking context, relative to a nonsmoking context.

Though the aforementioned result may indicate that the reward context does not moderate the relationship between smoker status and inhibitory control, there are alternative explanations. For instance, noting that a valid go/no-go task is a relatively fast-paced task, it may have been problematic that in one experimental block, both reward-related pictures and neutral pictures were randomly interleaved. This way, no relatively consistent and predictable reward context was created, which may have negatively impacted the ecological validity of the paradigm and effect size. Specifically, in the paradigm as implemented by Luijten et al. [3], participants did not know whether a given trial was reward related or not, and response bias to reward-related stimuli may have been reduced. Houben et al. [24] carried out a relatively similar task (stop signal task—SST) with a more stable reward context; the stimulus type (neutral/reward related) was separated across two conditions (a reward-related condition and neutral condition). This latter study supports the notion that inhibitory control (in this case, in individuals with obesity) varied as a function of the reward context. One may expect that the same applies to nicotine addiction because inhibitory deficits seem most pronounced in the context of reward.

The main aim of the current study was to assess the relation between nicotine addiction and inhibitory control in contexts that differed in terms of reward. We employed a go/no-go task modeled after version 4 as described by Wessel et al. [6]. The task consisted of neutral, smoking (smoking related pictures) and money (pictures of money) conditions. The following hypotheses were postulated. Firstly, we hypothesized that individuals that smoke, relative to people that do not smoke, would present with a reduced proportion of inhibitions in the smoking condition, relative to the neutral condition. Secondly, we hypothesized that this would also apply for the money condition, relative to the neutral condition. Thirdly, we hypothesized that the relative reductions in inhibitory performance would be mirrored by speeded responses to go trials.

## 2. Materials and Methods

### 2.1. Participants

We used convenience sampling, and participants were recruited via advertisements via various social media (most predominantly, Facebook). The advertisements included a link to the website that included the information letter, a link to provide informed consent, and a subsequent link to the experiment. The final sample consisted of 78 participants (55% male, 45% female). The mean age of the group that consisted of individuals that smoke (*n* = 43) was 28 years old (range 18–44, median = 27, *SD* = 6), and for the individuals that do not smoke (*n* = 35), the mean age was 23 years old (range 18–33, median = 23, *SD* = 3). Participants could not participate if they were using drugs (except nicotine) within seven days prior to participating. Participants had to be between 18 and 50 years old and healthy (by self-report). Participants could only be included in the smoker group in the case of smoking approximately 10 or more cigarettes per day in the past year. This inclusion criterion was based on several studies that have used the same or similar criterion [3,25,26]. The level of craving as assessed with the tobacco craving questionnaire, was overall relatively high (median = 72, *SD* = 14.9). Participants could be included in the nonsmokers group if they never smoked on a daily basis and if they did not smoke at all in the three months prior to participation [27]. Participants participated voluntarily and did not receive monetary compensation. The study was approved by the Research Ethics Committee of the of the Institute of Psychology, Eötvös Loránd University (ELTE) and conducted following the declaration of Helsinki.

### 2.2. Materials and Procedure

#### 2.2.1. Tobacco Craving Questionnaire (TCQ)

The short version of the TCQ instrument [28] assesses tobacco craving across four subscales (emotionality, expectancy, compulsivity, and purposefulness). Each of the four scales is measured with three items. Items are scored on a Likert scale, ranging from 1 (strongly disagree) to 7 (strongly agree). The final craving score is computed as the sum of all item scores. The final craving score can range from 12 to 84. Higher scores reflect a stronger craving. The subscales are known to show good reliability (Cronbach’s alpha: 0.90, 0.89, 0.78 and 0.69, respectively) [28].

#### 2.2.2. Go/No-Go Task

**Figure 1:**
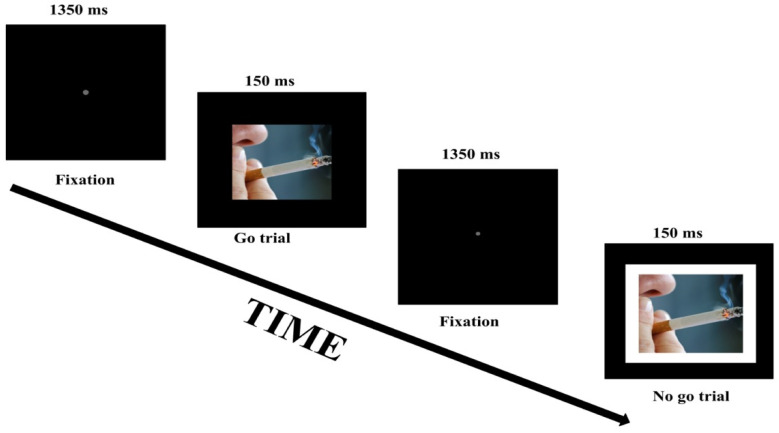
Schematic representation of two trials in the smoking-related condition of the go/no-go task.

The go/no-go task was employed to assess inhibitory control and was modeled after the go/no-go task (version 4) as described by Wessel et al. [6]. Each condition started with the instruction followed by a fixation dot, which was presented for 2000 ms. A single trial in each condition started with a go or no/go stimulus presented centrally for 150 ms, followed by a fixation dot presented for 1350 ms. Hence, the trial-to-trial interval was 1500 ms. Go stimuli (400 × 400 pixels) required a spacebar response, and no-go stimuli (500 × 500 pixels) were similar to the go stimuli but included a white border and required a response to be withheld. Each participant completed three separate conditions. In each trial in the neutral condition, the target stimulus (go or no-go stimulus) was one of four possible color filled squares. In each trial in the smoking condition, the target stimulus was one of four possible pictures related to smoking (Figure 1). For the money condition, the target stimulus consisted of one of four possible pictures of money. The pictures were in the public domain, and royalty free to use, download, copy, modify and adapt. The trials and condition order were randomized. For each condition, the probabilities of go and no-go stimuli were 0.8 and 0.2, respectively. The total number of trials per condition was 40. The conditions were preceded by a practice block consisting of 12 trials in which the target stimuli were gray squares. Inhibitory performance was indexed by the proportion of inhibitions to no-go trials. In other words, the proportion of inhibition was calculated as the number of successful non-responses to no-go stimuli divided by the total number of no-go trials. The response time was based on go trials only; and fast/accidental (<150 ms) and delayed responses (>1500 ms) were discarded from the analyses.

### 2.3. Procedure

The assessments were implemented online via Psytoolkit [29,30]. All participants were fully informed and signed the digital informed consent paper prior to participation. Subsequently, participants provided demographic information and performed the go/no-go task. The average total duration of the experiment was approximately 15 min.

### 2.4. Statistical Analyses

The sample size was determined using G*power [31,32] based on results provided by Luijten et al. [3]. Specifically, the main effect (η_p_^2^) of the group, regarding accuracy on the no-go trials (relevant index of inhibitory control), was estimated via F*(df1)/(F*(df2) + (df1)) [33]. η_p_^2^ was estimated to be 0.098, which translates to an f effect size of 0.33 [31,32]. With power set at 0.8, and alpha at 0.05, the optimal sample should be approximately *n* = 76. In total, 154 individuals expressed initial interest in participating (reflected by the number of clicks on the experiment link). However, the final sample size (*n* = 78) was lower, due to either not meeting the inclusion criteria or due to not completing the experiment. In addition, we excluded participants (*n* = 4) that had an omission rate that exceeded 3 standard deviations from the mean omission rate. Effectively, those participants were excluded with over 40% omissions, as this can indicate task non-adherence. For the remaining analyses, repeated measures ANCOVAs were performed, and we controlled for age differences. Specifically, age was included as a covariate.

## 3. Results

### 3.1. Smoking Status and Inhibitory Control across Conditions

**Figure 2:**
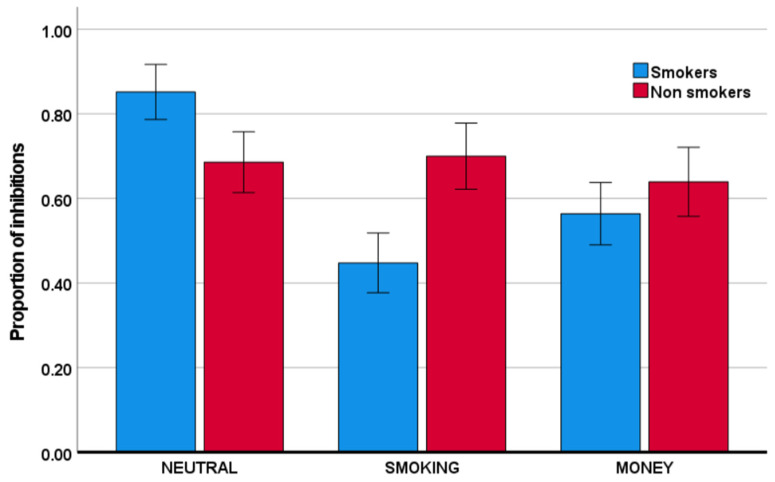
Mean proportion of inhibitions (the number of successful non-responses to no-go stimuli divided by the total number of no-go trials) for the smoker and nonsmoker groups across the neutral, smoking, and money conditions. Error bars indicate +/− 2 standard errors from the mean.

**Figure 3:**
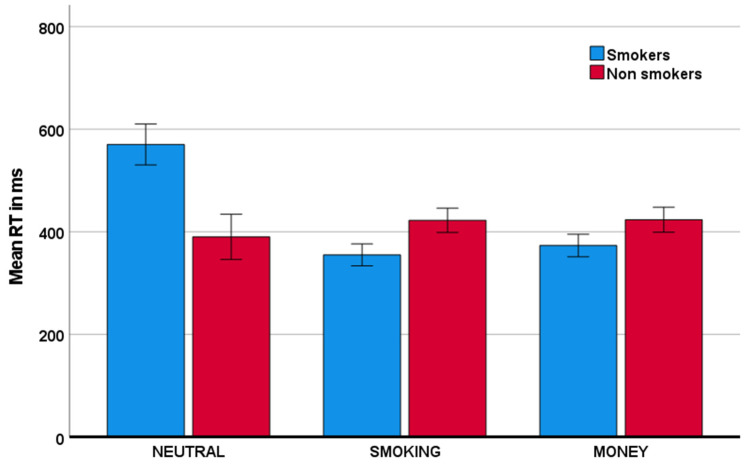
Mean reaction time (RT) for both smoker and nonsmoker groups across the neutral, smoking, and money conditions. Error bars indicate +/− 2 standard errors from the mean.

As is evident from Table 1 and Figure 2, the group of individuals that smoke relative to those that do not smoke showed a lower proportion of inhibitions in the smoking condition relative to the neutral condition. Specifically, this group x condition interaction was significant with F(1,75) = 22.08, *p* < 0.001, and partial η^2^ = 0.23. Similarly, smokers, relative to nonsmokers, also showed a reduced proportion of inhibitions in the money condition, relative to the neutral condition. This group x condition interaction was significant with F(1,75) = 7.31, *p* = 0.008, and partial η^2^ = 0.09. The reductions in inhibitory performance were mirrored by reductions in response time, described in Table 2 and visually depicted in Figure 3. Specifically, smokers, relative to nonsmokers, showed reduced response time in both the smoking condition and the money condition, relative to the neutral condition. These group x condition interactions were significant with, respectively, F(1,75) = 25.40, *p* < 0.001, and partial η^2^ = 0.25, and F(1,75) = 25.67, *p* < 0.001, and partial η^2^ = 0.26. With respect to the proportion of omissions (Table 3), there was no significant group x condition interaction (for both partial η^2^ < 0.003).

### 3.2. Inhibitory Performance and Response Speed in the Neutral, Smoking and Money Contexts as a Function of Craving in Smokers: An Ad Hoc Exploratory Analysis

**Figure 4:**
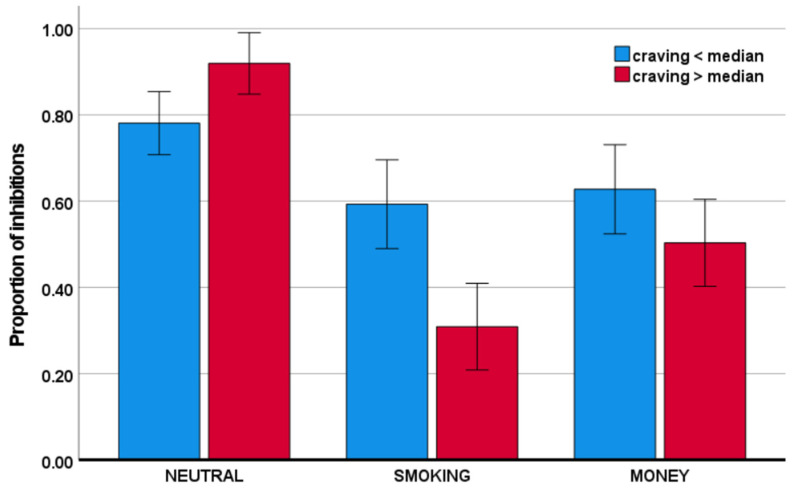
Proportion of inhibitions as a function of craving across conditions.

Craving was associated with a larger reduction in the proportion of inhibitions in the smoking condition, relative to the neutral, condition, as well as in the money condition, relative to the neutral condition (respectively, F(1,40) = 29.51, *p* < 0.001, η_p_^2^ = 0.425; F(1,40) = 9.21, *p* = 0.004, η_p_^2^ = 0.187). The effects are visualized in Figure 4. Please note that to visualize the direction of effects, craving was transformed to a categorical variable, using median split.

**Figure 5:**
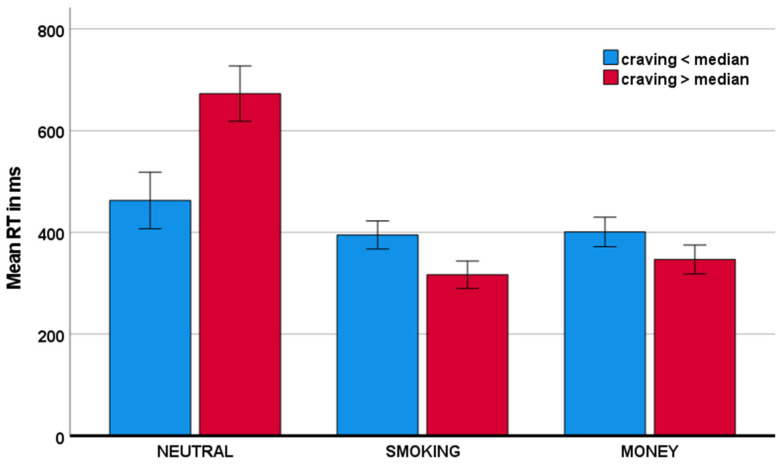
Mean response time as a function of craving across conditions.

With respect to the response time to go stimuli, the craving x condition interactions were also significant. Specifically, as visualized in Figure 5, craving was associated with reduced response time to smoking-related stimuli and money stimuli, relative to neutral stimuli: F(1,40) = 43.35, *p* < 0.001, η_p_^2^ = 0.520; and F(1,40) = 36.16, *p* < 0.001, η_p_^2^ = 0.475, respectively.

It should be noted that the distribution of craving scores showed a significant negative skew, with skewness = −1.720. Although it is known that analyses of variance are relatively robust against deviations from normality, we reran the above analyses on normalized data, using a common log transform to the inverse distribution. Specifically, we subtracted all scores from the maximum score +1 in the distribution and subsequently applied the log transform. The repeated measures ANCOVAs did not result in a different outcome regarding the significance of the contrasts (data are available in a publicly available repository).

## 4. Discussion

Our results showed that individuals that smoke, relative to those that do not smoke, have reduced inhibitions in smoking and money contexts, relative to a neutral context. Importantly, response time analyses showed that individuals that smoke, relative to those that do not smoke, responded slower in the neutral context, relative to the reward contexts.

It should be noted that the smokers’ relatively lower number of inhibitions in both reward contexts as compared to the neutral context may also be (at least in part) due to the slowed responses in the neutral context. Specifically, the response time data indicate that individuals that smoke may adopt a waiting strategy in the neutral context and engage in faster responding in both reward contexts, which may at least contribute to reduced inhibitory performance. Smokers’ relatively speeded responses in reward contexts is congruent with IS theory [18] as well as with Volkow’s dopamine motive model as outlined in the introduction [17]. In short, IS theory predicts a higher approach or response bias in those individuals afflicted by nicotine addiction to stimuli that have reward value, which in turn may contribute to poorer inhibitory performance in such contexts, which is exactly what our results suggest.

Importantly, the lower inhibitory performance in the money condition relative to the neutral condition in individuals that smoke may indicate a generalized reduced inhibitory control in any context of reward, or alternatively/additionally, an increased response bias. Interestingly, these performance effects seem to extend to other contexts of learned rewards, such as the context of money.

Further explorative analyses regarding inhibitory control as a function of craving also seem in line with what would be expected from IS theory and the dopamine motive model. In particular, to the extent that tobacco craving is associated with heightened motivational tendencies and associated attentional bias as well as reduced inhibition toward cigarette-associated stimuli, the same would be predicted for other reward-related stimuli. Indeed, our results indicate that craving in smokers is associated with challenged inhibitory control and enhanced attentional bias in relation to both smoking-related cues as well as money-related cues, relative to a neutral context.

It may be surprising that the results of Luijten et al. (2011) did not support a reward context–specific inhibitory deficit in individuals that smoke, relative to individuals that do not smoke. There was quite some overlap between our study and the one from Luijten et al. in terms of the paradigm and in- and exclusion criteria. However, one important difference was the operationalization of a reward context. In contrast with our study and the one from Houben et al. (which also showed that inhibitory control varied as a function of the reward context, albeit in individuals with obesity), the reward context was not stable, and relatedly, reward-related targets were not predictable. Specifically, no separate conditions were implemented, and an experimental run included both reward-related and neutral pictures. One other important difference was that individuals in the study from Luijten et al. [3] were not allowed to smoke within an hour prior to participation, which might have induced some heightened craving, which might explain the more general reduction in inhibitory control. A slightly different interpretation is that acute nicotine-induced cognitive enhancement in the present study may have induced proactive slowing; however, in the reward conditions, this is counteracted by the enhanced approach tendency.

Indeed, the slowed responses in the neutral condition in individuals that smoke is mirrored by a more than two-fold higher standard deviation, relative to the other conditions. Though at first glance this might reflect the presence of outliers, we should emphasize that there were no extreme response times characterized as values exceeding three standard deviations from the mean. Hence, the larger response time variability indicates higher individual differences in the smoker group in terms of response speed.

It should be emphasized that the exact brain mechanism that explains the effects on inhibitory control and speed of responding is yet to be elucidated. Certainly, the proportion of inhibitions as assessed in the implementation of the go/no-go task in our study reflects (at least partly) inhibitory control and is associated with an electrophysiological (brain activity) measure of inhibition [6]. Even so, other cognitive brain processes most plausibly contribute to the observed behavioral effects. For instance, congruent with IS theory, addicted individuals may present with enhanced attentional bias for reward-related stimuli. It follows that when attentional bias and related approach behavior are sizeable for a no-go stimulus that is related to reward, inhibitory control is challenged. Based on performance measures alone, it is difficult, if not impossible, to disentangle the relative contributions of attentional processes from inhibitory processes. Combining the go/no-go paradigm with brain activity measures of attention and inhibitory control may address this unanswered question and provide more information on the neurophysiological and neuroanatomical correlates of involved attentional and inhibitory processes.

Another limitation concerns the stimuli used for the induction of reward-related activity. Smoking-related pictures were based on Luijten et al. [3] and money stimuli were based on our previous report [34]. However, though it is very plausible, based on previous studies, that reward-related activity is induced by the reward-related stimuli (with the possible exception of smoking-related stimuli in relation to nonsmokers) [17,18], we do not know the degree of activation as a function of an exact stimulus. Hence, for future studies, it would be interesting to complement the behavioral measures with brain activity measures of reward processes to further scrutinize the exact role of reward processing in the observed behavioral effects. Pertaining to the latter, one suggested interesting approach would be to implement a reward prediction violation task that includes the different reward contexts and combine it with fMRI and/or EEG. During this task, reward-predicting stimuli are presented, which are sometimes followed by an unexpected consequence. Importantly, it was shown using such tasks that reward delivery and reward prediction are driven by different neural mechanisms [35]. How these mechanisms operate in nicotine addiction across different conditions of reward and drive behavioral performance remains an open question.

A somewhat related limitation of the current study is that the conditions were not matched on stimulus complexity and the stimuli in the reward contexts may be viewed as more complex; thus, one might argue that our observed effects reflect, at least partly, the interaction between smoker status and stimulus complexity. However, the response time results contrast such a notion. To elaborate, significantly higher stimulus complexity would yield increased response times. Yet, no increased response time in the reward contexts, relative to the neutral context, was observed. In fact, for individuals that smoke, response times decreased significantly in the reward contexts. Taken together, it is not plausible that the observed effects are due to the interaction of smoker status with stimulus complexity.

The age restriction could also be seen as a limitation. The main reason for the age limit was based on recent studies that suggest that inhibition mechanisms might be affected by aging, especially around and over the age of 60 [36,37]. To avoid any such potential age-related effects, we employed a strict age restriction. Of course, one may argue that as age progresses in individuals that smoke, their exposure to nicotine and associations also increases. That could be the case, but then the observed group x condition effects would most plausibly be even more (not less) pronounced.

Lastly, one might argue that the online implementation of the study is a limitation. It was questioned whether data can be acquired reliably in online cognitive psychological experiments that require fast split-second logging of event durations and response times. However, recent studies have shown that online experiments, including those that have stringent timing criteria, can record valid and reliable data that are comparable to data acquired in labs using dedicated hardware and software [38,39]. Similarly, and specific to the Psytoolkit platform, the results of previous studies indicate that experiments with stringent timing criteria for logging data can generate reliable and valid data [30,39]. Of course, that does not mean that online experiments do not include any challenges. Individuals performing an online experiment cannot ask for additional information as easily as in a lab environment, and task understanding and adherence may be challenged. In our study, we took this into account and used the proportion of omissions as an index of task adherence. To elaborate, omissions to go stimuli are quite rare in a fast-paced go/no-go task [23], and hence, we excluded those participants who displayed a relatively high rate of omissions. Lastly, we believe that the online format effectively increased ecological validity while reducing common biases. Pertaining to the latter, desirability and acquiescence bias are deemed minimal, due the anonymous nature and due to the implemented objective measures together with a lack of information about how the constructs are reflected in actual responses. Order bias may affect condition effects; however, any potential/plausible order effects are controlled for via counterbalancing. Certainly, sampling bias is a challenge in almost any type of empirical research, including online studies. However, we tried to minimize sampling bias by means of advertising across a wide variety of different social media channels, and not focusing on one isolated social media group.

## 5. Conclusions

Our results suggest that individuals that smoke, as compared to nonsmokers, have reduced inhibitory performance in a smoking context, which may extend to other learned reward contexts. The reduced inhibitory performance may be due to the speeded responses in these conditions, indicating increased reward-related response bias. Taken together, the results seem consistent with IS theory and cross sensitization. In terms of clinical implications, our results may imply that challenged inhibitory control in pharmacological addiction and craving may extend to other reward contexts. This may be considered in treatment approaches.

## Figures and Tables

**Table 1:** Proportion of inhibitions to no-go trials for smokers and nonsmokers in the neutral, smoking, and money conditions.

Condition	Group	Mean	Std. Deviation
neutral	smokers	0.85	0.19
	nonsmokers	0.69	0.24
smoking	smokers	0.45	0.27
	nonsmokers	0.70	0.18
money	smokers	0.56	0.24
	nonsmokers	0.64	0.25

Note: Proportion of inhibitions: the number of successful non-responses to no-go stimuli divided by the total number of no-go trials.

**Table 2:** Mean response time in milliseconds in go trials for smokers and nonsmokers in the neutral, smoking, and money conditions.

Condition	Group	Mean	Std. Deviation
neutral	smokers	570	165
	nonsmokers	390	68
smoking	smokers	355	74
	nonsmokers	422	65
money	smokers	373	71
	nonsmokers	423	74

**Table 3:** Proportion of omissions in go trials for smokers and nonsmokers in the neutral, smoking, and money conditions.

Condition	Group	Mean	Std. Deviation
neutral	smokers	0.02	0.04
	nonsmokers	0.02	0.05
smoking	smokers	0.03	0.05
	nonsmokers	0.03	0.08
money	smokers	0.02	0.04
	nonsmokers	0.02	0.03

## Data Availability

Data are publicly available at the Open Science Framework (osf.io), DOI 10.17605/OSF.IO/D2NZ8.
